# Limb Salvage Surgery in a Voluminous Malignant Quadriceps Tumor

**DOI:** 10.7759/cureus.45717

**Published:** 2023-09-21

**Authors:** Muhammad Z Farooq, Muhammad B Shafiq, Ilyas Rafi, Sajid Ali

**Affiliations:** 1 Surgical Oncology, Shaukat Khanum Memorial Cancer Hospital and Research Centre, Lahore, PAK; 2 Paediatric Surgical Oncology, Shaukat Khanum Memorial Cancer Hospital and Research Centre, Lahore, PAK

**Keywords:** tumor surgery, secondary malignancy, quadriceps tumor, limb salvage, sarcoma

## Abstract

It is uncommon for soft tissue sarcomas to develop after adenocarcinoma of the rectum. In a treated rectal adenocarcinoma patient, we encountered a huge malignant quadriceps tumor as leiomyosarcoma and salvaged the limb.

A 49-year-old male known case of treated moderately differentiated adenocarcinoma of the rectum presented in the Orthopedic Clinic with a new swelling in his left thigh. MRI of the left lower limb was obtained, and it demonstrated a large 15.8 x 13.2 x 30 cm well-defined mixed solid cystic lesion in the anterolateral aspect of the left mid-thigh without the involvement of adjacent bony cortex. Limb salvage surgery with wide local excision of the tumor was done. The patient was ambulated full weight from the very next day with a Musculoskeletal Tumor Society Score (MSTS) score of 20.

Despite the massive size of the tumor, limb salvage was attempted successfully and achieved good functional status.

## Introduction

Extremity soft tissue sarcomas are uncommon, heterogeneous malignant tumors that make up less than 1% of all cancers [1]. The thigh is the most common site of presentation in extremity soft tissue sarcoma [2]. Historically, amputation was the standard for local disease control, but over the last few decades, there has been a shift towards limb salvage due to the evolution of multimodal therapy [3]. The anatomical location of the sarcoma defines the functional outcome after limb salvage surgery [4]. These tumors usually respect the fascial boundaries and therefore it is possible to resect the whole compartment with clear resection margins even if the tumors are large [5]. Soft tissue sarcomas are treated with an adequate wide local excision followed by adjuvant therapy (chemoradiation). In the case of malignant tumors of the quadriceps, the strength of extension at the knee joint is reduced depending on the extent of excision of the quadriceps and preservation of any muscle group [6]. However, for large quadriceps malignant tumors, it is sometimes necessary to resect the whole of the quadriceps to obtain clear margins resulting in loss of extension at the knee joint. This can result in loss of strength in the lower limb, but will provide a functional limb in comparison to the loss of limb. The resulting defect can be compensated by free flap transfer of the latissimus dorsi to the quadriceps [7]. The following case is an example of oncological resection of enormous quadriceps leiomyosarcoma to salvage the limb and management of functional loss with a knee brace. Malignant soft tissue sarcomas presenting as a second malignancy in rectal adenocarcinoma patients are rare.

## Case presentation

**Figure 1:**
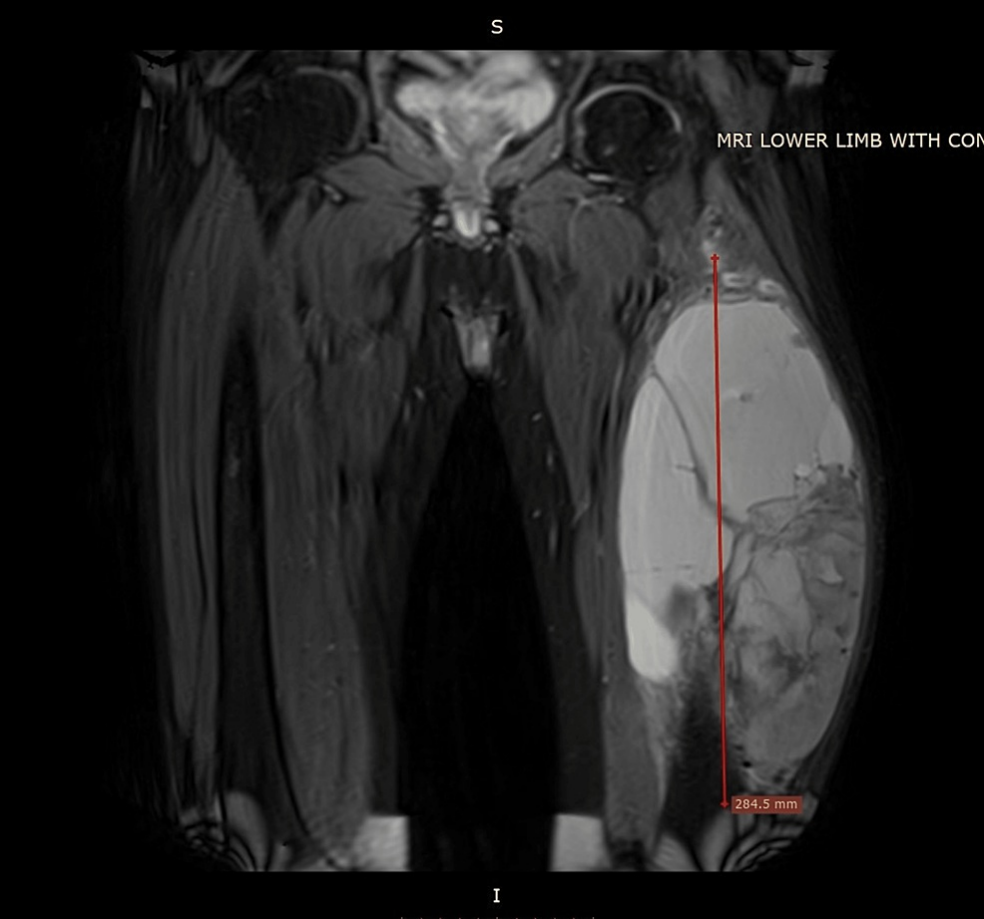
Shows the T1 MRI coronal dimensions of the tumor.

**Figure 2:**
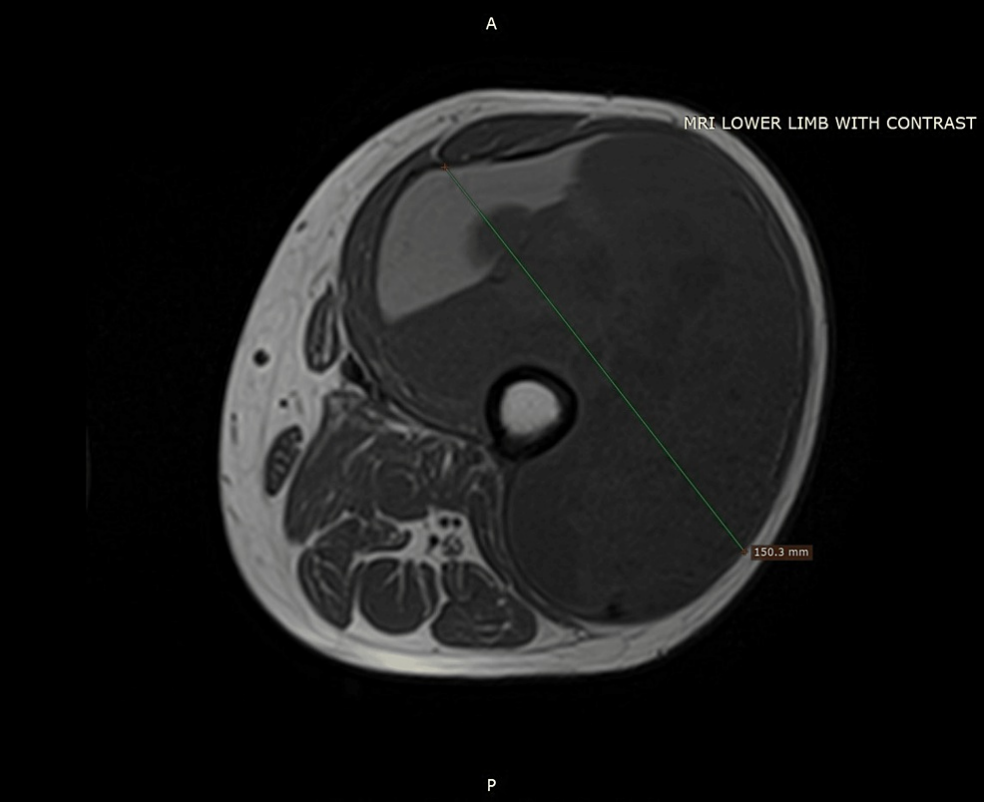
Shows the T1 MRI axial dimensions of the tumor involving anterolateral compartment of thigh.

A 49-year-old male known case of treated moderately differentiated adenocarcinoma of the rectum presented in the Orthopedic Clinic with a new swelling in his left thigh. Before this presentation, the patient had received neoadjuvant chemotherapy followed by laparoscopic anterior resection and adjuvant chemotherapy. The patient was disease-free and on regular oncological follow-up for one year when he noticed a new swelling and pain in his left thigh. MRI of the left lower limb was obtained, and it demonstrated a large 15.8 x 13.2 x 30 cm well-defined mixed solid cystic lesion in the anterolateral aspect of the left mid-thigh without the involvement of adjacent bony cortex. This lesion involved the vastus intermedius, vastus lateralis, vastus medialis, and rectus femoris, and had several thin internal septations with an internal hemorrhagic component. Figures 1, 2 show the findings of T1 coronal and axial views of MRI.

The neurovascular bundle was intact. No size-significant inguinal or popliteal lymphadenopathy was noted. No other lesion was noted.

A Trucut biopsy of the thigh lesion was performed, and it was revealed to be leiomyosarcoma Grade 2 as a second malignancy. The case was discussed in our routine clinic-pathological conference and the decision was taken to perform a wide local excision of the tumor with intent of limb salvage. Elective surgery of the patient was planned after counseling him about the potential loss of limb extension.

**Figure 3:**
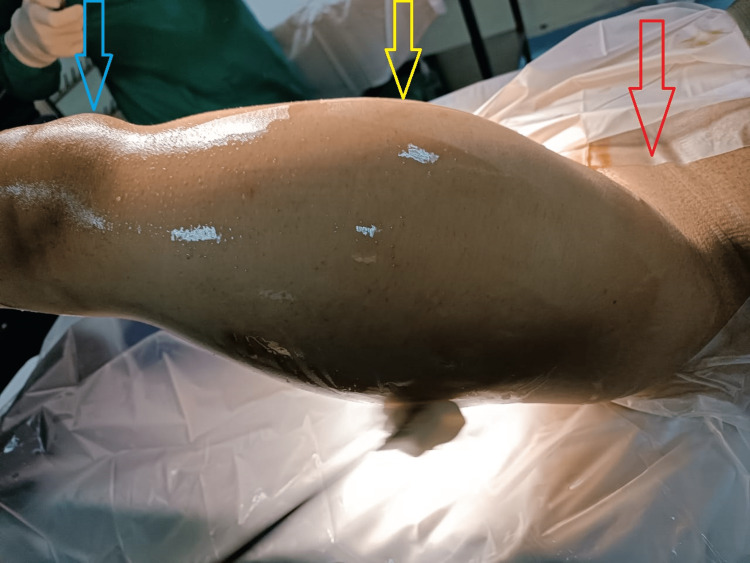
Clinical presentation of the tumor at the time of surgery. Red arrow shows anterior superior iliac spine, yellow arrow shows the tumor mass involving the anterolateral compartment of thigh and blue arrow shows patella.

An elliptical incision was made over the swelling extending from the anterior superior Iliac spine to the lateral border of the patella (Figure 3). Skin and subcutaneous tissues were dissected, the medial skin flap was raised and the femoral nerve and vessels were identified and separated from the tumor.

**Figure 4:**
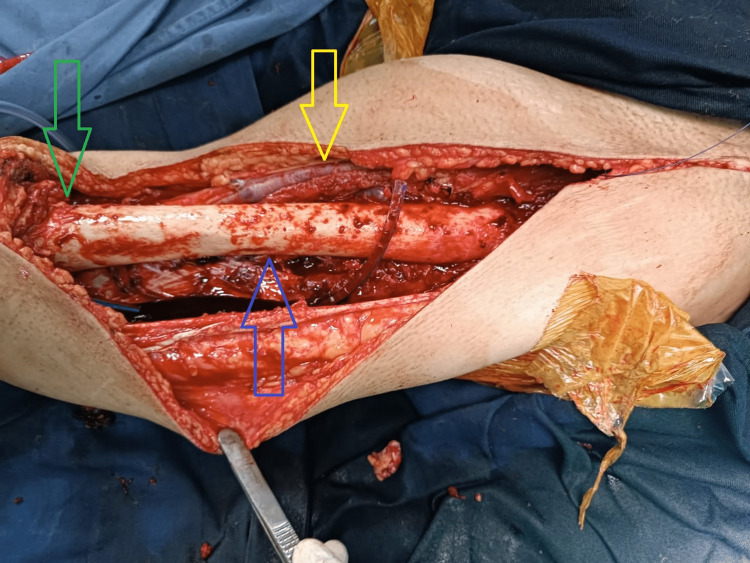
Post resection tumor bed. Yellow arrow shows femoral artery and vein. Blue arrow shows femur bone. Green arrow shows proximal pole of patella.

**Figure 5:**
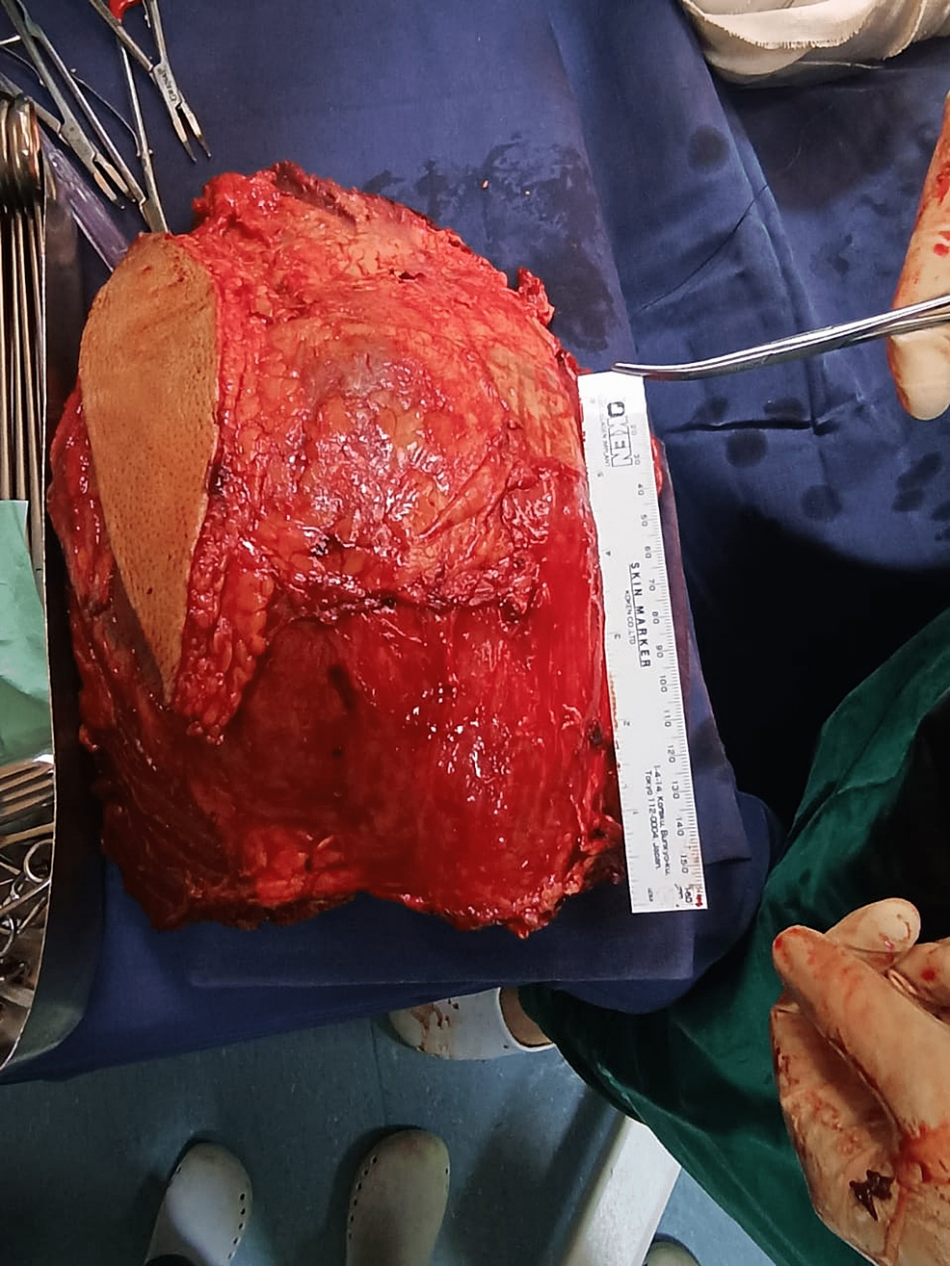
Tumor after resection.

The profunda femoris artery was ligated and an en bloc-wide local excision of the whole tumor was performed. Superiorly quadriceps muscle attachments from the iliac bone were separated and inferiorly muscles were resected at the level of quadriceps tendon attachment on the patella. The tumor was attached to the femur and excised along with its periosteal attachments (Figure 4). The length of the final tumor resection was measured at 30 cm (Figure 5).

The patient was ambulated full weight-bearing with the help of a knee brace from the first postoperative day. Post-operative histopathology showed it to be grade 3 leiomyosarcoma as per Federation Nationale Des Centres De Lutte Control Le Cancer (FNCLCC) [8] with clear resection margins of more than 5mm and involvement of medial margin.

The case was re-discussed in a clinic-pathological conference and a decision of adjuvant radiotherapy was made. The patient received adjuvant radiotherapy 66 Gray, 33 fractions, three weeks after surgery.

At 14 months follow-up the patient is completely pain-free and ambulates with the help of a knee brace. The Musculoskeletal Tumor Society Score (MSTS) of the patient is 20 [9].

## Discussion

Soft tissue sarcomas are a heterogeneous group of sarcoma with above 50 subtypes. Leiomyosarcoma is one of the most common subtypes. The treatment of leiomyosarcoma is particularly difficult due to the variability of its biological origin, clinical behavior, and chemosensitivity, as well as the dearth of effective clinical studies [10]. For this reason, the treatment for leiomyosarcomas has to be individualized. Leiomyosarcoma as second malignancy has been reported in a small pediatric population [11] but has not been reported in adult patients who have already been treated for rectum adenocarcinoma. Rizwan et al. reported a huge leiomyosarcoma as primary malignancy of the posterior compartment thigh, treated with wide local excision and salvaged the limb [12]. Surgical resection of the leiomyosarcoma is the most common treatment, followed by radiation to eradicate any residual disease [13]. Post excision there are multiple reconstruction options available, but a good functional result can be achieved with the help of a brace and adequate rehabilitation; this has been evident in our case as well as the one reported by Rizwan et al. [12].

This kind of cancer has an unknown etiology, and the risk factors have not yet been discovered. Cancer symptoms can vary depending on the cancer's location and stage, but common ones including fatigue, fever, and weight loss are frequently observed. Although swelling or a lump is frequently recognized because tumors typically grow extremely quickly, local pain is rare [3,5]. The tumor also affects the ambulatory status of the patient depending on the muscle compartment involved as seen in our case [7].

Modalities like CT scan and MRI are being used to diagnose leiomyosarcoma of the thigh with multiple distinguishing features [14] but the final diagnosis is made on the basis of histopathology and its grade according to FNCLCC [8]. The higher the grade of tumor, the poorer the prognosis. Metastasis is ruled out on the basis of bone scan and CT thorax and treatment is individualized [4]. Although non-uterine leiomyosarcomas can metastasize to the brain as reported by Oka et al. [15] due to the rarity of such a spread, brain workup is usually not included in the baseline workup of leiomyosarcoma unless symptoms warrant that.

Soft tissue leiomyosarcomas are quite aggressive tumors. Patients with leiomyosarcoma have only a chance of 50% three-year survival [16]. Combined with the presence of another malignancy treated previously increases the morbidity and decreases the chances of survival, although no proper evidence is yet present in the literature. As our patient has already been treated for rectal adenocarcinoma there is a probability of any genetic malformation present in such a patient. Gene analysis is not being done in this patient due to lack of proper diagnostic facilities. We recommend gene pool studies in patients with rectal adenocarcinoma presenting with second malignancies.

## Conclusions

Leiomyosarcoma can present in a variety of locations with an array of symptoms. Leiomyosarcomas presenting as a second malignancy in rectal adenocarcinoma patients is rare. Even the primary cases of thigh leiomyosarcomas that have been reported are rare. There has been no well-defined treatment protocol for such a patient. Nevertheless, an attempt should be made to salvage the limb involved with leiomyosarcoma through surgical resection with wide margins. Despite being a massive tumor involving the whole quadriceps muscle, we salvaged the limb and achieved good functional status on ambulation with a knee brace. Although multiple reconstruction options are considered with muscle flaps to restore the function of the excised limb compartment, any such intervention in a patient with multiple malignancies would have increased the morbidity and an equivalent functional outcome has been achieved with the knee brace. We conclude that such tumors shall be treated with limb salvage surgeries whenever possible, as this improves the functional outcome and survival of the patient.
